# The development of an automated sentence generator for the assessment of reading speed

**DOI:** 10.1186/1744-9081-4-14

**Published:** 2008-03-28

**Authors:** Michael D Crossland, Gordon E Legge, Steven C Dakin

**Affiliations:** 1 UCL Institute of Ophthalmology, London, UK; 2 University of Minnesota, Minneapolis, MN, USA

## Abstract

Reading speed is an important outcome measure for many studies in neuroscience and psychology. Conventional reading speed tests have a limited corpus of sentences and usually require observers to read sentences aloud. Here we describe an automated sentence generator which can create over 100,000 unique sentences, scored using a true/false response. We propose that an estimate of the minimum exposure time required for observers to categorise the truth of such sentences is a good alternative to reading speed measures that guarantees comprehension of the printed material. Removing one word from the sentence reduces performance to chance, indicating minimal redundancy. Reading speed assessed using rapid serial visual presentation (RSVP) of these sentences is not statistically different from using MNREAD sentences. The automated sentence generator would be useful for measuring reading speed with button-press response (such as within MRI scanners) and for studies requiring many repeated measures of reading speed.

## Background

The assessment of reading is of continued interest to those working in many areas of science including ophthalmology, the neurosciences and developmental psychology. Difficulty in reading is the most frequent complaint of people with acquired eye disease attending low vision clinics [1-4], and reading ability can predict performance on other daily skills such as watching television, detecting faces and using kitchen utensils [5]. Although comprehension or reading age are the most common measures of reading performance in education, in vision science and the neurosciences in general, reading speed is the most common metric of reading. [6] Reading speed is sensitive to visual factors such as text size, contrast and sampling density [7,8] and is significantly reduced in visual impairment [9].

A variety of tests of reading speed are available. In vision science, perhaps the most frequently used technique is the MNREAD test [6,10]. This consists of a standardised set of sentences, of equal length, complexity and word structure, which are presented in gradually decreasing size. Plotting reading speed against print size creates a distinctive function with a large plateau of peak reading speed and a decline for text presented at very small or very large print sizes.

Although the MNREAD test has been shown to be rigorous and repeatable [11,12], it does have certain limitations. One disadvantage of the MNREAD test is that comprehension is not assessed: subjects are able to repeat the sentences without any understanding of the meaning of the words which are read. Tests of reading for understanding (*"rauding"*) involve the addition of simple comprehension questions which are answered after subjects have read a passage of text [13-15]. As these questions can frequently be answered without reading every single word of the text, some redundancy may be present within each passage of words. So-called "speed readers" may develop a strategy of skipping words to read only the words likely to appear on a comprehension test. An alternative approach might be to ask subjects to read simple mathematical expressions, and then ask them to respond "true" or "false" to questions such as "2 + 4 = 6" (true) or alternatively "three times two equals seven" (false). The advantage of such an approach is that the test can be fully automated with observers making their response using the computer keyboard. The disadvantage if that there is a high degree of redundancy in such sentences: given the limited corpus of words which can realistically be used, a skilled observer could rely solely on the first one or two characters of each word in order to answer the statement. Furthermore, the comprehension required for this task is very different than understanding a weather forecast or enjoying a novel, for example. A final approach to guaranteeing comprehension within a reading test is to score simple sentences as true or false, such as the method used by Just [16] and in educational attainment tests such as the Neale analysis of reading ability test [17-19].

A disadvantage of all reading tests is that they all have a reasonably limited corpus of sentences, words or paragraphs. Those requiring an oral response rely on an investigator being present and scoring the number of words read correctly, and can not be administered in an environment where movement is limited, such as a fMRI scanner or some eyetrackers.

Our aim was to develop an automated sentence generator to create an almost unlimited corpus of sentences, with no redundancy, which can be scored with a simple true/false judgement by the observer to allow for full automatization of measurement. Here we describe such a system which produces, at random, a 4 word sentence, with no redundancy, with a true/false response. We compare reading speed functions assessed with this system to MNREAD sentences.

## General methods

### The sentence generator

The sentence generator was written in Matlab (v. 7.0.4, Mathworks, Natick, MA) by SCD. It consists of a corpus of words arranged into three categories: quantifiers, objects and descriptions. Each sentence is constrained to have the following structure:

Quantifier | Noun | Two word description

**Figure 1:**
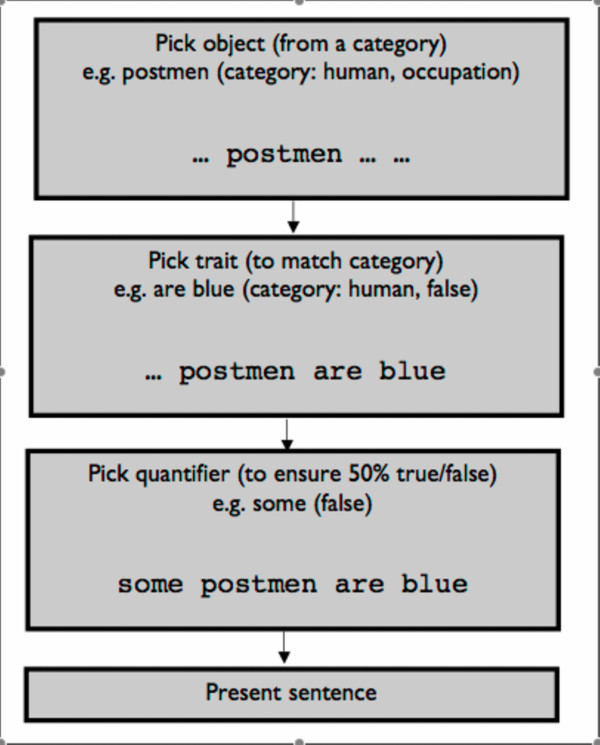
Schematic describing the structure of the sentence generator.

First, a noun is selected at random from one of 414 words currently in the corpus (such as "architects", "penknives", "ale" or "music"). Second, a grammatically appropriate two-word description (which may be true or false) is selected from a set constrained to the item or category in question: for example, a human trait ("read books"), a non-living-object trait ("don't breathe") or a specific trait ("design buildings", "aren't sharp", "is jazz"). A schematic of the sentence generation algorithm can be seen in figure 1. One hundred sample sentences are given in the additional information [Additional file 1].

At present there are over 1,000 unique descriptions in the database, although not all descriptions can be applied to all nouns. Finally a quantifier is chosen from the set "no", "some" or "all". The combination of quantifier and description is selected such that half of all of the sentences are true. Double negative sentences (e.g. "No alligators can't read") are prohibited. We conservatively estimate that this creates around 100,000 unique allowed sentences. The Flesh-Kincaid grade level of 500 randomly selected sentences was found to be 3.6.

### Subjects

Nine normally sighted observers participated in the experiments described below. All were under the age of 40 (mean age 26 years; range 21–35 years), read English fluently and were educated to University degree level. Eight subjects read English as a first language (four were British, three Irish, one Australian) and one (S2) was a native Cantonese speaker with fluency in English. Four were female. No subjects had any ophthalmological or neurological disease, and all had visual acuity (with corrective lenses if required) of 6/6 (20/20) or better. One of the authors (MDC) was an observer in experiment 1 but not subsequent experiments due to his familiarity with the MNREAD sentences used. No other subjects were familiar with the stimuli used. The study conformed to the tenets of the Declaration of Helsinki and subjects gave their informed consent prior to data collection.

### Stimulus presentation

Stimuli were created on an Apple computer using custom functions written in Matlab (v.7.3; Mathworks, Natick, MA) based on elements of the Psychophysics toolbox [20,21] and were presented on an 19" CRT monitor (ElectronBlue II; Lacie, Massy, France) with a 60 Hz refresh rate.

Automatically generated sentences were created at random using the sentence generator. A different random seed was used for each experiment so that each observer read different sentences. MNREAD sentences were taken from a corpus of 525 such sentences (supplied by Dr Elisabeth Fine, previously of Harvard Medical School) all of which followed the construction rules defined in the original MNREAD specification [22]. In order to ensure equivalent demands on memory, only the first 4 words of each MNREAD sentence were presented.

Sentences were presented in black Courier font against a white background. Courier is a fixed-width font in which each character occupies an equal amount of horizontal space. Four different text sizes were used during the experiment: with *x*-height of 0.1°, 0.3°, 1°, 3°. Subjects viewed the larger three sizes from 50 cm and the smaller words from 2 m.

## Experiment 1

As some of the generated sentences are cognitively demanding, this experiment was performed to determine the proportion of sentences scored as correct under completely unconstrained conditions. The "lapse rate" was identified as being the number of sentences incorrectly scored under conditions with no visual or temporal constraints.

The second aim of this experiment was to ensure that every word in the sentence must be processed for a true/false judgement to be made accurately. A lack of redundancy ensures that subjects are not able to "cheat" on the test by using a strategy of only attending to certain words to make their decision. If our sentences genuinely have no redundancy then removing any word will drop performance to near chance (i.e. 50%). This validation experiment ascertains the effect of removing any word by presenting the text with no time pressure and asking subjects to determine whether the sentence is true or false from the information given.

### Method

Six observers (including S1, one of the authors) participated in this experiment. Sentences were presented, one sentence at a time, on the monitor. Sentences were presented only at an *x*-height of 1°, in black Courier font on a white background.

In condition one, stimuli were presented as complete (four word) sentences. Observers were asked to judge whether the sentence was true or false, and to respond by means of a button press. No time constraint was imposed on the task.

Condition two was identical although one randomly selected word was missing from the sentence, such that only three words were presented. If the fragment made sense (e.g. from the sentence "some dogs have legs", if the fragment was "dogs have legs") observers were asked to judge whether the sentence was true or false based on the information given (true in this example). However, if the fragment was nonsensical ("all dogs legs") then participants were asked to select true or false at random.

Each observer viewed at least 120 sentences under each of the conditions (3 words/4 words), in interleaved blocks of 60 trials. The proportion of sentences scored correctly in each block was recorded.

### Results

The proportion of correct responses is shown in table 1. Under the unconstrained condition, between 2% and 17% of sentences were incorrectly scored. Removing any word at random from the sentence significantly reduced comprehension (from a mean value of 92% to 58%; Wilcoxon sign-rank test: *p *< 0.05). Removing word three had significantly less effect on comprehension than removing any other word (to a mean of 72%; Tukey HSD test: *p *< 0.05). There is no statistical difference between removing word 1, 2 or 4 from the sentence: removing each of these words reduces performance to a mean value of 54% (Tukey HSD test: p > 0.05).

**Table 1:** The effect on comprehension of removing one word at random from the sentences.

	% of sentences read correctly					
	
Subject	No word removed	Any word removed	Word 1 removed	Word 2 removed	Word 3 removed	Word 4 removed
S1	95	56	50	50	67	58
S2	83	64	60	66	68	63
S3	92	59	55	53	65	62
S5	88	59	55	65	73	57
S8	98	54	36	49	79	50
S9	97	57	36	51	79	63

### Discussion

Removing any word from the sentence reduces performance on an identification task. If any word other than the third word is removed, this performance is near chance, whereas if word 3 is removed performance drops to around 73%. This difference can be attributed to two-word phrases in the second half of the sentence being relatively easy to guess (if the third word in the sentence "No grapes like music" is deleted, it is comparatively easy to realise that there is no relationship between grapes and music, and therefore the sentence is correctly scored as true).

Some three word sentences will convey the correct meaning. If any one of the permutations does make sense, and subjects guess at 50% for the remaining three (nonsensical) sentences, the expected proportion of correct answers will be 62.5%. This may explain why performance does not fall to exactly 50%. It should be noted that even if the three-word reduced sentence makes sense, the meaning may be incorrect. For example, if the sentence "no sheep are electric" (true) was displayed with word 1 missing, the display would be "sheep are electric" (false).

We conclude that whilst our sentences are not completely free of redundancy, as performance falls significantly with the absence of one word, only a minimal level of redundancy exists within these sentences.

## Experiment 2

The purpose of experiment 2 was to compare reading speed assessed using sentences created by the sentence generator to a standard and widely used reading speed test (the MNREAD test). In addition, we compared two methods of scoring the generated sentences: first, by an investigator recording how many words per sentence were read correctly, and second by the observer grading the sentences as true or false.

### Method

**Figure 2:**
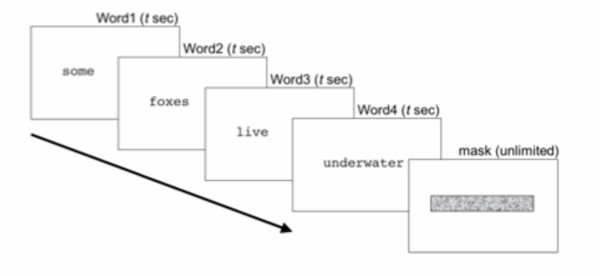
**Experiment design. **Each word is presented in sequence for *t *seconds, followed by a mask of dynamic noise, presented for an unlimited period of time.

Seven observers (Subjects S3–S9) participated in this experiment. Subjects 1 and 2 were not tested due to their knowledge of all of the MNREAD sentences. Reading performance was assessed using the rapid serial visual presentation paradigm (RSVP) whereby each word is presented individually, one after the other, in the same screen position (figure 2) [23-25]. The benefit of RSVP is that no eye movements are needed during the reading process, such that extremely fast reading speeds can be obtained.

#### Sentence presentation

Sentences were presented in black courier font against a white background. Peak luminance of the screen was 125 cdm^-2^. Subjects viewed the monitor from 2 m for text sizes of less than 1° and 50 cm for other conditions. A dynamic noise mask was presented following the last word of the sentence to counteract iconic memory.

To measure reading speed, the psychophysical method of constant stimuli (MCS) was used. For each sentence, the MCS presents each word for a fixed duration. The durations chosen were randomly selected from 1, 2, 3, 5, 8, 12, 19, and 30 frames (corresponding to 0.016, 0.033, 0.05, 0.083, 0.13, 0.2, 0.32 and 0.5 seconds respectively, or 3,600 to 120 words/minute). Within each block, six sentences were presented at each exposure duration, so 48 sentences were presented per block.

In order to construct a reading speed function, text was presented at four text sizes (text with *x-*height of 0.1°, 0.3°, 1° and 3°). Text size was constant within each block.

Word duration was interleaved between sentences, but within each sentence each word was presented for the same length of time. In order to measure a reading speed function, text was presented at four sizes: of *x-*height 0.1°, 0.3°, 1°, and 3°. Text size was constant for each block of sentences. Figure 2 shows the experimental design.

Three conditions were used. In the first condition, the sentence generator presented a randomly produced four-word sentence, and the subject was asked to read the sentence aloud. The investigator scored the number of words read correctly (between 0 and 4) by comparing the verbal report to the sentence (displayed in easily legible print on a supplementary laptop screen observable only by the investigator). Credit was given for words read correctly but in the wrong order. Stimulus presentation was identical in condition 2, but observers were asked to describe each statement as being true or false. Following text presentation, the response time was unconstrained, and participants were encouraged to think for as long as necessary before responding. In condition 3, the four words presented were the first four words of a randomly chosen MNREAD sentence. Subjects were asked to report the words aloud, and were aware that the sentence fragment may not make sense in isolation. The number of words read correctly was recorded by the observer in the same manner as condition 1.

Following several practice trials to ensure that subjects were familiar with the task and with the nature of the sentences, each observer performed 12 blocks of sentences (three conditions **X **four text sizes) in a randomly determined order. Within each block there were 48 sentences (six sentences **X **eight exposure durations). Participants were encouraged to take breaks between blocks.

The proportion of correct responses was plotted as a function of exposure duration to create a psychometric functions for every observer under each condition and text size. Reading speed was defined as 0.8 correct from the psychometric function.

### Results

#### Reading speed

**Figure 3:**
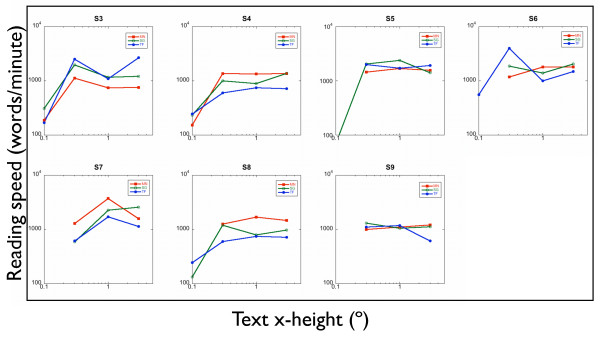
**RSVP reading speed as a function of text size for five observers.** SG(0–4): Sentence generator sentences, scored as 0–4 correct. SG(T/F): Sentence generator sentences, scored as true or false. MNREAD: MNREAD sentence fragments (4 words long), scored as 0–4 correct.

Figure 3 shows reading speed as a function of text size for the three conditions: MNREAD sentences (red curves, legend MN), sentence generator sentences scored by marking the number of words read correctly (green curves, SG) and the sentence generator sentences with true/false scoring (bue curves, TF in the legend). Reading speed was determined as being the speed corresponding to the 80% point on the psychometric function for each condition. Each curve shows the characteristic decline in reading speed for the smaller text. Note that some subjects did not reach 80% performance at the smallest text size.

**Figure 4:**
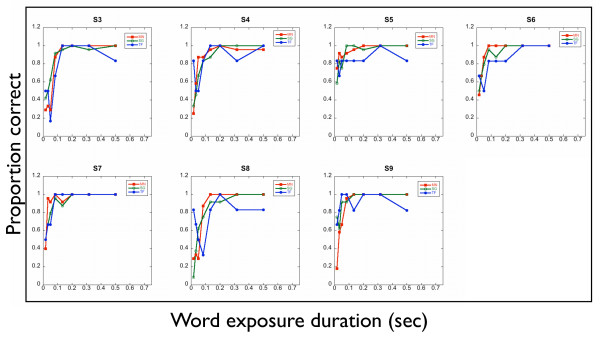
**Psychometric functions of reading speed for five observers at *x*-height of 1°. **SG(0–4): Sentence generator sentences, scored as 0–4 correct. SG(T/F): Sentence generator sentences, scored as true or false. MNREAD: MNREAD sentence fragments (4 words long), scored as 0–4 correct.

No systematic difference is observed in peak reading speed between the three conditions (Wilcoxon signed-rank test: MNREAD *v *sentence generator with true/false scoring: *p *= 0.81; MNREAD *v *sentence generator scored by number of words correct: *p *= 0.58) or between the two methods of scoring the generated sentences (Wilcoxon signed-rank test: true/false *v *number of words correct: p = 0.08). Full psychometric functions for reading text under the three conditions for text size of 1° are shown in figure 4 and table 2. It can be seen that no systematic difference was observed between the three methods of assessing reading.

**Table 2:** Reading speed using each presentation method for each observer at x-height of 1°.

**Observer**	**Reading speed at 1°**		
	
	**MNREAD**	**Automated (n correct)**	**Automated (true/false)**
S3	744	1162	1100
S4	1338	890	748
S5	1700	2397	1720
S6	1799	1400	999
S7	3719	2279	1707
S8	1686	798	748
S9	1102	1056	1188

## General discussion

We have developed an automated system to create sentences which can be answered with a simple true/false response. The automatically generated sentences have minimal redundancy and comprehension is required to correctly score them as being true or false. The reading speed function produced using the generated sentences is qualitatively similar to that measured using the MNREAD test, and these sentences give similar peak reading speed results to the MNREAD test.

This system can be used in a stand-alone fashion without the observer reading sentences aloud, making it possible to measure reading speed under conditions where speaking is not possible, such as within a MRI scanner. A staircase procedure could easily be added to measure reading speed or to maintain stimulus presentation near threshold for functional imaging of the brain whilst reading. The true/false response given by the participant also removes the potential source of error of an investigator determining whether words are read correctly or not. The very large number of possible sentences would be of particular benefit for longitudinal studies or those where repeated measures of reading speed are required.

There are a few caveats to our reading speed test. First, participants must be instructed to accept statements at face value and not to over-analyse the sentences before responding true or false. For example, the sentence "all dogs have legs" would be scored as true, although perhaps a very small number of dogs may have no legs. Similarly, participants had to be informed that "most" was not exclusive of "all": "most children were born" is scored as true even though on a semantic level it may not be strictly accurate. Despite careful instruction, we assume that these confusions contributed to the high lapse rate indicated in table 1. Second, although the (rather simplistic) Flesch-Kincaid reading age of our sentences suggests they could be read by a young child, some of the sentences have quite difficult meanings: consider "no snakes are mammals" (true) or "some bakers are mortal" (true). It is important to note that our software is written such that editing the word lists used is straightforward: they could easily be altered to be appropriate for assessing children, for example.

The MNREAD test has been designed to only include words from the 2,000 most frequently occurring words in American English. The sentence generator corpus was not written with a specific word frequency in mind. Post-hoc analysis of forty-eight randomly generated sentences was performed to identify the frequencies fo words appearing. 62% of the words were in the 2,000 most frequent words in British English, with only 18% being outside the top 6,000 words [26]. Low frequency words included atheist, butchers, panthers, sparrows and cheetahs. In each condition, each subject read 24 sentences at each exposure duration and 48 sentences at each text size; and for every observer the sentences were different and selected at random. It is extremely unlikely that word frequency effects would have accounted for the word-size effects which we show in figure 3, or the psychometric functions in figure 4. To confirm this, a post-hoc analysis of 48 sentences with randomly assigned exposure durations was performed. This confirms that there is no relationship between exposure duration and word frequency (r^2 ^< 0.00001). However, in subsequent experiments using fewer sentences, word frequency effects could be controlled by editing the corpus.

Finally, when pilot testing was performed in the laboratory of GEL, it was found that some cultural references were missed by those who had never lived in Europe (for example, "voting conservative" had no meaning; and a Peugeot was not identified as a car). Again, editing of the database can eliminate this problem.

A further limitation of our sentences is that they are only 4 words long and so would fit on one line: page reading (involving retrace to the start of subsequent lines and page navigation) can not be assessed easily using such short sentences. This is in contrast to MNREAD sentences which are typically presented over three lines.

Our data on a small number of observers is not enough to establish this test as being comparable to the MNREAD cards or any other test of reading speed, and in this methodological paper we do not aim to suggest that this is the case. A Bland-Altman analysis of at least 50 participants is required to accurately determine the limits of repeatability and variability of any clinical technique [27]. Rather, we aim to show in this manuscript that an automated sentence generator can be used to produce intelligible sentences which can be scored using a dichotomous true/false outcome and which produce reading speed functions qualitatively similar to those created with a more traditional test such as MNREAD.

## Conclusion

We have developed a Matlab based automated sentence generator which can produce over 100,000 unique sentences. The sentences have little redundancy, require comprehension, and can be scored with a simple true/false response. The sentence generator can be easily expanded to increase or limit its vocabulary. We anticipate that this technique will be of particular use for experiments of reading in experiments using fMRI, and in longitudinal studies requiring repeated measures of reading.

## Abbreviations

RSVP: Rapid serial visual presentation; MRI: Magnetic resonance imaging; fMRI: Functional magnetic resonance imaging; MCS: Method of constant stimuli; MN: MNREAD sentences; SG: Sentence generator sentences, scored using the number of words read correctly; TF: Sentenced generator sentences, scored using a true/false response.

## Competing interests

The author(s) declare that they have no competing interests.

## Authors' contributions

MDC recruited participants, collected and analysed the data and wrote the first draft of the manuscript. GEL assisted in experimental design and data analysis. SCD conceived, developed and adapted the sentence generator programme. All authors contributed to, read and approved the final manuscript.

## Supplementary Material

Additional file 1 Further example sentences. One hundred randomly selected phrases created by the sentence generator are given, along with the correct response (t: true; f: false). Click here for file
