# Supplementary material for: The development of an automated sentence generator for the assessment of reading speed
Source: Behav Brain Funct. 2008 Mar 28;4:14. doi: 10.1186/1744-9081-4-14 (PMC2294132; doi:10.1186/1744-9081-4-14)
Supplement: Additional file 1 — Further example sentences. One hundred randomly selected phrases created by the sentence generator are given, along with the correct response (t: true; f: false). [file 1744-9081-4-14-S1.doc]

some hawks are reptiles (f)

some budgies aren't mammals (t)

no carpenters are cats (t)

no celebrities are insects (t)

some mackerel can sing (f)

some apartments have floors (t)

some actors are thoughtful (t)

some foxes have lungs (t)

no bears have brains (f)

some Volkswagens are silver (t)

no butlers like football (f)

no bears are warm-blooded (f)

no nurses can swim (f)

some bakers were born (t)

no sharks are fish (f)

some telephones like theatre (f)

some vicars have bodies (t)

some comedians eat nothing (f)

some celebrities were born (t)

no strawberries are sweet (f)

some hamsters are iron (f)

some butlers are plants (f)

no babies like milk (f)

no countries can walk (t)

some policemen wear wings (f)

no bankers can fly (t)

some priests are republican (t)

no moles are fish (t)

no barmen drink water (f)

no carpenters hate rugby (f)

some people live underwater (f)

some bankers are electronic (f)

some bakers are fish (f)

no engineers have paws (t)

some poplars drive cars (f)

no gorillas have brains (f)

no detectives have antlers (t)

no secretaries are atheists (f)

no antelope have eyes (f)

some offices have tails (f)

some Fords need servicing (t)

some musicians can fly (f)

all shepherds never die (f)

no Renaults have airbags (f)

some shepherds are generous (t)

no chimps have feathers (t)

all bakers aren't religious (f)

some ravens have bones (t)

no minibuses are vehicles (f)

no teachers are female (f)

some shepherds are considerate (t)

some oaks have roots (t)

some hotels are meat (f)

no politicians live underwater (t)

no bakers are fish (t)

all panthers aren't reptiles (t)

no universities can read (t)

some detectives like football (t)

no tomatoes are juicy (f)

some comedians have fur (f)

no giraffes can fly (t)

no lawyers are mean (f)

no gerbils have legs (f)

no budgies have eyes (f)

no students are democrats (f)

no shops are sheep (t)

some swans haven't eyes (f)

some detectives have feathers (f)

no Fords have seatbelts (f)

no lions are mammals (f)

all dogs aren't male (f)

some shops are inanimate (t)

no Peugeots have boots (f)

some butchers are republican (t)

no people have feathers (t)

no Vauxhalls can laugh (t)

some bankers read newspapers (t)

no ducks have gills (t)

some comedians are unemployed (t)

no butchers are cotton (t)

no comedians hate rugby (f)

some Saabs breathe air (f)

no politicians are unemployed (f)

no sheep are insects (t)

no elephants are green (t)

some maids have fins (f)

no houses can read (t)

no builders have paws (t)

some homes read novels (f)

no trout have fins (f)

some politicians can breathe (t)

some politicians are independent (t)

some elephants are grey (t)

no diaries type fast (t)

some gorillas are insects (f)

no gin is alcoholic (f)

some moose have gills (f)

no engineers are religious (f)

no engineers live underwater (t)

no pears are blue (t)
